# Western and Eastern experience in treating perihilar cholangiocarcinoma: retrospective bi-centre study

**DOI:** 10.1093/bjsopen/zraf019

**Published:** 2025-04-09

**Authors:** Hannes Jansson, Atsushi Oba, Aya Maekawa, Christina Villard, Kosuke Kobayashi, Yoshihiro Ono, Jennie Engstrand, Fumihiro Kawano, Hiromichi Ito, Stefan Gilg, Yosuke Inoue, Melroy A D’Souza, Yu Takahashi

**Affiliations:** Division of Surgery and Oncology, Department of Clinical Science, Innovation and Technology, Karolinska Institutet, Stockholm, Sweden; Division of Hepatobiliary and Pancreatic Surgery, Cancer Institute Hospital, Japanese Foundation for Cancer Research, Tokyo, Japan; Division of Hepatobiliary and Pancreatic Surgery, Cancer Institute Hospital, Japanese Foundation for Cancer Research, Tokyo, Japan; Division of Hepatobiliary and Pancreatic Surgery, Cancer Institute Hospital, Japanese Foundation for Cancer Research, Tokyo, Japan; Division of Transplantation Surgery, Department of Clinical Science, Innovation and Technology, Karolinska Institutet, Stockholm, Sweden; Division of Hepatobiliary and Pancreatic Surgery, Cancer Institute Hospital, Japanese Foundation for Cancer Research, Tokyo, Japan; Division of Hepatobiliary and Pancreatic Surgery, Cancer Institute Hospital, Japanese Foundation for Cancer Research, Tokyo, Japan; Division of Surgery and Oncology, Department of Clinical Science, Innovation and Technology, Karolinska Institutet, Stockholm, Sweden; Division of Hepatobiliary and Pancreatic Surgery, Cancer Institute Hospital, Japanese Foundation for Cancer Research, Tokyo, Japan; Division of Hepatobiliary and Pancreatic Surgery, Cancer Institute Hospital, Japanese Foundation for Cancer Research, Tokyo, Japan; Division of Surgery and Oncology, Department of Clinical Science, Innovation and Technology, Karolinska Institutet, Stockholm, Sweden; Division of Hepatobiliary and Pancreatic Surgery, Cancer Institute Hospital, Japanese Foundation for Cancer Research, Tokyo, Japan; Division of Surgery and Oncology, Department of Clinical Science, Innovation and Technology, Karolinska Institutet, Stockholm, Sweden; Division of Hepatobiliary and Pancreatic Surgery, Cancer Institute Hospital, Japanese Foundation for Cancer Research, Tokyo, Japan

## Abstract

**Background:**

Resection outcomes for perihilar cholangiocarcinoma differ between Western and Eastern centres, but reasons behind these disparities remain unclear. This study aimed to compare current outcomes between a Western and an Eastern expert centre to identify prognostic factors.

**Methods:**

Patients who underwent hepatobiliary resection for perihilar cholangiocarcinoma between 2010 and 2022 at Karolinska University Hospital (Stockholm, Sweden) and Cancer Institute Hospital (Tokyo, Japan) were retrospectively included. Primary outcome was overall survival. Secondary outcomes were disease-free survival, postoperative complications and 90-day mortality rate.

**Results:**

Two hundred and forty-nine patients were included (Cancer Institute Hospital *n* = 159, Karolinska *n* = 90). Median overall survival was 20.4 months at Karolinska and 52.0 months at Cancer Institute Hospital (*P* < 0.001). Median disease-free survival was 11.9 months at Karolinska and 32.4 months at Cancer Institute Hospital (*P* < 0.001). Advanced tumours, ASA class ≥III, poor differentiation and radial margin positivity were more common in the Western cohort. Treatment centre, T-status, N1-status, resection side, R1-status, age and carbohydrate antigen 19-9 were prognostic for overall survival. The Eastern cohort had a lower rate of postoperative complications (24.5%) and a lower mortality rate (2.5%) compared with the Western cohort (51.1% and 10.0%).

**Conclusion:**

Advanced tumour stage and radial margin positivity contributed to poor long-term survival in the Western cohort. A higher burden of co-morbidity and a higher rate of extended resections with smaller remnant liver volume influenced the Western postoperative mortality rate.

## Teaser

This study analysed current outcomes after resection for perihilar cholangiocarcinoma in a large bi-institutional Western–Eastern cohort to identify prognostic factors. Advanced tumour stage and radial margin positivity were found to contribute to poor long-term survival in the Western cohort. A higher burden of co-morbidity and a higher rate of extended resections with smaller remnant liver volume influenced the Western postoperative mortality rate.

## Introduction

Despite advances in care over the past decades, the postoperative morbidity rate following resection for perihilar cholangiocarcinoma (pCCA) is considerable, and long-term outcomes remain unsatisfactory^1^. Combined hepatic and biliary resection has enhanced the prospects for radical resection and long-term survival, as illustrated by Nagoya University (Japan)^2,3^. With the introduction of preoperative portal vein embolization (PVE) to allow augmentation of the future liver remnant (FLR), more extensive resections can be undertaken while minimizing the risk of posthepatectomy liver failure (PHLF)^4,5^. Only in recent years, phase III trial data has started to accrue to support the use of adjuvant chemotherapy for patients with pCCA^6,7^.

Ever since the landmark publication by Tsao *et al*., which compared the experience from Nagoya with early experiences at an American centre (Lahey)^3^, enduring differences in Western and Eastern outcomes have been evident^8,9^. Kimura *et al*. showed disparities in morbidity and mortality rates between Leeds (UK) and Hirosaki (Japan)^9^, and Olthof *et al*. identified lower overall survival (OS) rates in a multicentric Western cohort (USA, The Netherlands) compared with Hokkaido (Japan)^8^. Mueller *et al*. recently found lower OS when comparing Western to Eastern centres^1^. The degree to which differences in long-term outcomes could be explained by variations in surgical–oncological strategy, demographics or tumour characteristics remains unclear. Notably, previous reports indicating survival differences lacked data on oncological therapy^1,8^. This study aimed to compare patient and tumour characteristics, surgical–oncological therapy and postoperative short- and long-term outcomes after pCCA resection at a Western and an Eastern expert centre.

## Methods

### Study design

This study included consecutive patients who underwent hepatobiliary resection for histopathologically verified pCCA between 1 January 2010 and 15 May 2022 at Karolinska University Hospital (Stockholm, Sweden) and the Cancer Institute Hospital (CIH) of the Japanese Foundation for Cancer Research (Tokyo, Japan). Data were collected retrospectively from institutional registries and electronic medical records. While at Karolinska the electronic records were updated from the national population registry for vital status, at CIH regular telephone follow-up with referring centres and patient/family to confirm vital status was carried out. At Karolinska, patients were followed after surgery with blood samples and computerized tomography every 6 months for 3 years, and then yearly for up to 5 years after surgery. At CIH, patients were followed after surgery with blood samples and computerized tomography every 3 months for 2 years, and then every 6 months up to 5 years after surgery. Patients with a macroscopically tumour positive resection margin (R2), isolated extrahepatic bile duct resection or synchronous hepatobiliary and pancreatic resection were excluded from this analysis. The study was approved by the institutional ethical review boards (Regional Ethical Review Board Stockholm 2015/259–31/2; Swedish Ethical Review Authority 2022-06962-02; JFCR 2023-GB-128) and reported according to STROBE guidelines^10^.

### Outcome variables and clinicopathological data

The primary outcome variable was OS (calculated from the date of surgery). Secondary outcome variables were disease-free survival (DFS)^11^, PHLF grade B or C and bile leakage grade B or C, according to the International Study Group of Liver Surgery (ISGLS) criteria^12,13^, postoperative complications Clavien–Dindo grade III or higher^14^ and postoperative 90-day mortality rate.

The following demographic and clinicopathological data were collected: age, sex, body mass index (BMI), carbohydrate antigen 19-9 (CA19-9) concentration, American Society of Anesthesiologists (ASA) physical status classification, diagnosis of primary sclerosing cholangitis (PSC), Bismuth–Corlette classification^15^, preoperative biliary drainage, preoperative PVE, FLR volume, histopathological tumour extension (T), lymph node metastasis (N1), lymphatic invasion (L1), microvascular invasion (V1), perineural invasion (Pn1) and microscopic tumour positive resection margin (R1)^16^. The type and extent of resection was documented, with major resection defined as involving three or more hepatic segments and extended resection as left or right trisectionectomy according to the ‘New World’ terminology^17^. A definition of R0 as a tumour-free margin (> 0 mm) was used for this study^16^, as this was the standard at CIH. Data for Karolinska using a ≥ 1 mm tumour-free margin definition have been previously presented^18^. The proximal, distal and radial margin status was registered. Pathological grade was reported according to the College of American Pathologists guidelines^16^, and pathological staging according to the AJCC/TNM 7th edition, as it was the standard in use for a majority of the time interval studied^19^. At Karolinska, the examination of the surgical specimen was performed according to a detailed institutional protocol^18^. At CIH, the evaluation of the surgical specimen was performed in accordance with the Japanese classification of biliary tract cancer guidelines^20^.

Receipt of neoadjuvant or adjuvant chemotherapy was registered. During the study period, both institutions used gemcitabine (alone or in combination with cisplatin) or an oral fluoropyrimidine (Karolinska: capecitabine; CIH: S-1). Capecitabine was introduced into clinical routine at Karolinska after results from the BILCAP (capecitabine compared with observation in resected biliary tract cancer) trial (2019)^6^, while at CIH adjuvant S-1 was introduced with the JCOG1202/ASCOT (adjuvant S-1 compared with observation in resected biliary tract cancer) trial (2013–2018) and thereafter according to the JCOG1202/ASCOT protocol^7^. Preceding these phase III trials, observation only was the standard of care at both institutions, with adjuvant chemotherapy reserved for selected patients with high-risk characteristics, for example younger patients with lymph node metastasis or a positive resection margin.

### Preoperative evaluation and planning

At both institutions, clinical diagnosis and preoperative staging was undertaken by a multidisciplinary tumour board. At both centres, biliary decompression by the endoscopic route was recommended as first-line intervention when indicated, with drainage of the intrahepatic duct of the future remnant lobe and with additional drainage catheters inserted as necessary to control jaundice and cholangitis. Whereas at CIH, decompression was primarily performed by nasobiliary drainage or with subsequent suprapapillary plastic stent^21,22^, at Karolinska, transpapillary plastic stent decompression was routinely used^23^. Percutaneous transhepatic biliary drainage was reserved as a rescue procedure for patients not attaining successful drainage by endoscopic retrograde cholangiography. On the basis of computerized tomography after biliary decompression, three-dimensional reconstruction was performed for liver volumetry. While all patients at CIH underwent liver volumetry, volumetry was performed selectively at Karolinska (for example before planned right-sided resection). Volume modulation was performed by PVE to reach a target FLR of at least 40% at CIH and at least 30% at Karolinska. Portal vein embolization was performed after sufficient biliary decompression, by use of microparticles (polyvinyl alcohol, trisacryl gelatin) with proximal coil or plug occlusion (Karolinska) or absolute ethanol (CIH). Hypertrophy of the FLR was evaluated with computerized tomography 2–3 weeks after PVE at both institutions.

### Statistical analysis

Categorical variables are reported as numbers and percentages, continuous variables as medians with interquartile ranges (i.q.r.). The chi-square or Fisher’s exact tests were used to compare proportions, as appropriate. The Mann–Whitney *U* test was used for the comparison of distributions. Survival was analysed using the Kaplan–Meier method with log-rank test and univariable and multivariable Cox regression. Variables with a univariable association at *P* < 0.200 were included in multivariable analysis. Proportional hazards were tested with time-dependent covariates. Receipt of adjuvant therapy was modelled as a time-dependent variable to account for the unexposed survival time^24,25^. Median follow-up was calculated using the reverse Kaplan–Meier method^26^. Demographic and clinicopathological variables were reported with missing data. Multiple imputation was used for independent variables in regression analyses (10 imputed data sets). Sensitivity analyses were performed with complete case analysis. Predictor variables for imputation included all imputed variables and regression outcome variables. Statistical analyses were performed using SPSS® Statistics v. 28 (IBM, New York, USA). A two-tailed *P* value <0.05 was considered statistically significant.

## Results

### Demographic and clinical characteristics

Two hundred and forty-nine patients were included, 159 from CIH and 90 from Karolinska. The overall median follow-up time was 68.1 months, 64.7 months for patients from CIH and 80.2 months for patients from Karolinska (*P* = 0.070). The clinical characteristics of the two cohorts are presented in *Table 1*. The proportion of patients with preoperative ASA class ≥III was higher at Karolinska (*n* = 30, 33.3% *versus n* = 25, 15.8%, *P* = 0.001), whereas patients at CIH were older (*P* < 0.001) and had a lower median BMI (*P* < 0.001).

**Table 1:** Clinical characteristics

	Missing data	CIH *n* = 159	Karolinska *n* = 90	*P*
Age (years), median (i.q.r.)	–	71 (64–75)	63.5 (51–70)	<0.001*
**Sex**				**0.004**
Male		117 (73.6)	50 (55.6)	
Female		42 (26.4)	40 (44.4)	
ASA class ≥III	*n* = 1	25 (15.8)	30 (33.3)	**0.001**
BMI, median (i.q.r.)	–	21.8 (19.9–23.9)	24.1 (21.4–28.7)	<0.001*
CA19-9 (U/ml), median (i.q.r.)	*n* = 50	58.3 (11.6–255.7)	178.5 (47.8–757.3)	0.003*
PSC, yes	–	0	13 (14.4)	**<0.001** ^†^
**Bismuth–Corlette class**	*n* = 7			**<0.001** ^†^
I		20 (12.6)	3 (3.3)	
II		25 (15.7)	7 (7.8)	
IIIa		40 (25.2)	47 (52.2)	
IIIb		42 (26.4)	14 (15.6)	
IV		32 (20.1)	12 (13.3)	
**Biliary drainage**	–			**<0.001**
None		28 (17.6)	23 (25.6)	
EBD		110 (69.2)	44 (48.9)	
PTBD		18 (11.3)	7 (7.8)	
EBD + PTBD		3 (1.9)	16 (17.8)	
PVE		88 (55.3)	29 (32.2)	**<0.001**
Right-sided resection	–	72 (45.3)	58 (64.4)	**0.004**
Extended resection	–	24 (15.1)	60 (66.7)	**<0.001**
Segment 1 resected	–	159 (100.0)	70 (77.8)	**<0.001**
**Vascular resection**	–			**0.002** ^†^
PVR		30 (18.9)	25 (27.8)	
HAR+/−PVR		19 (12.0)	0	
**T extension**	*n* = 4			**<0.001** ^†^
T1		12 (7.7)	0	
T2a		67 (43.2)	16 (17.8)	
T2b		32 (20.6)	39 (43.3)	
T3		19 (12.3)	27 (30.0)	
T4		25 (16.1)	8 (8.9)	
N1	*n* = 7	64 (41.3)	50 (57.5)	**0.016**
L1	*n* = 14	97 (61.4)	67 (87.0)	**<0.001**
V1	*n* = 7	115 (72.8)	66 (78.6)	0.324
Pn1	*n* = 6	140 (88.6)	83 (97.6)	**0.014**
Grade ≥2	*n* = 8	102 (66.2)	84 (96.6)	**<0.001**
R1	*n* = 1	55 (34.8)	34 (37.8)	0.639
R1 proximal	*n* = 6	30 (19.5)	16 (18.0)	0.773
R1 distal	*n* = 6	26 (16.9)	4 (4.5)	**0.005**
R1 radial	*n* = 6	17 (11.0)	20 (22.5)	**0.017**
Preoperative chemotherapy	–	7 (4.4)	3 (3.3)	1.000^†^
Adjuvant chemotherapy	*n* = 5	44 (27.7)	26 (30.6)	0.631

Values are *n* (%) unless otherwise indicated. *P* < 0.05 in bold type. ASA, American Society of Anesthesiologists; BMI, body mass index; CA19-9, carbohydrate antigen 19-9; CIH, Cancer Institute Hospital; EBD, endoscopic biliary drainage; Grade ≥2, moderate or poor tumour differentiation; HAR, hepatic artery resection; i.q.r., interquartile range; L1, lymphatic invasion; N1, lymph node metastasis; Pn1, perineural invasion; PSC, primary sclerosing cholangitis; PTBD, percutaneous transhepatic biliary drainage; PVE, portal vein embolization; PVR, portal vein resection; R1, microscopically tumour positive resection margin; T ≥ 3, tumour extension status 3 or 4; V1, microvascular invasion. * Mann–Whitney *U* test. † Fisher’s exact test.

There was a significant difference in the distribution of preoperative Bismuth–Corlette classes between the cohorts. At Karolinska, a majority of operated patients had tumours classified as Bismuth–Corlette IIIa (*n* = 47, 52.2% *versus n* = 40, 25.2% at CIH, *P* < 0.001).

### Preoperative interventions

Comparing preoperative interventions, endoscopic biliary drainage was used in the majority of patients at both centres, while patients at Karolinska were more likely to undergo percutaneous biliary drainage (alone or in combination with endoscopic drainage, *n* = 23, 25.6% *versus n* = 21, 13.2% at CIH, *P* < 0.001). The proportion of patients with preoperative PVE was higher at CIH compared with Karolinska (*n* = 88, 55.3% *versus n* = 29, 32.2%, *P* < 0.001). While PVE was more common at CIH before both right hemihepatectomy (*n* = 66, 95.7% *versus n* = 4, 40.0% at Karolinska, *P* < 0.001) and left trisectionectomy (*n* = 20, 95.2% *versus n* = 0, 0% at Karolinska, *P* < 0.001), rates were similar at the two centres before right trisectionectomy (*n* = 2, 66.7% CIH *versus n* = 25, 52.1% Karolinska, *P* = 1.000). The median preoperative FLR was larger at CIH, 49.3% (i.q.r. 41.7–62.8) compared with 31.4% (i.q.r. 26.6–37.5) at Karolinska (*P* < 0.001). In patients undergoing PVE, the FLR differed significantly between centres, both before and after PVE (median pre-PVE FLR 21.9% Karolinska *versus* 31.5% CIH, *P* < 0.001; median post-PVE FLR 28.4% Karolinska *versus* 43.5% CIH, *P* < 0.001).

### Resection types

The type and extent of resection differed between centres (*Table 1*). Right-sided resections were performed more often at Karolinska (*n* = 58, 64.4% *versus n* = 72, 45.3% at CIH, *P* = 0.004). Trisectionectomy was more frequently undertaken at Karolinska compared with CIH (*n* = 60, 66.7% *versus n* = 24, 15.1%, *P* < 0.001). While similar overall proportions of patients underwent vascular resection, arterial resections were performed in 12.0% of all patients at CIH (*n* = 19) but were not employed at Karolinska (*P* = 0.002).

### Oncological therapy

Adjuvant systemic chemotherapy was used in similar proportions of patients at both centres, 27.7% (*n* = 44) at CIH and 30.6% (*n* = 26) at Karolinska (*P* = 0.631). Few patients received preoperative chemotherapy, 4.4% (*n* = 7) of patients at CIH and 3.3% (*n* = 3) at Karolinska (*P* = 1.000, *Table 1*).

### Postoperative histopathological stage and grade

Patients at Karolinska had higher rates of T2b and T3 tumours (*P* < 0.001), lymph node metastasis (*P* = 0.016), lymphatic invasion (*P* < 0.001) and perineural invasion (*P* = 0.014). Furthermore, well differentiated tumours were less common in the Karolinska cohort (*P* < 0.001, *Table 1*). Among patients with similar T-status, rates of lymph node metastasis did not differ significantly between centres (T1–T2a *P* = 0.160; T2b *P* = 0.689; T3–T4 *P* = 0.355, *Table S1*), while the histopathological grade differed within all T-status subgroups (grade ≥ 2 within T1–T2a *P* = 0.002; grade ≥ 2 within T2b *P* = 0.003; grade ≥ 2 within T3–T4 *P* = 0.002, *Table S1*).

### Lymph node count

The number of retrieved lymph nodes differed between centres, with a median number of 6 lymph nodes (i.q.r. 4–8) at Karolinska compared with 15 (i.q.r. 11–19) at CIH (*P* < 0.001). Still, the median number of positive lymph nodes was higher at Karolinska: one (i.q.r. 0–2) positive node compared with zero (i.q.r. 0–1) at CIH (*P* = 0.043).

### Radicality of resection

While the overall frequency of R1 was similar between centres (*n* = 34, 37.8% Karolinska *versus n* = 55, 34.8% CIH, *P* = 0.639), a radial R1 margin was more frequent in patients at Karolinska (*P* = 0.017) and a distal R1 more frequent at CIH (*P* = 0.005) (*Table 1*).

### Resection type and radicality according to Bismuth–Corlette class

The types of resections performed, stratified by preoperative Bismuth–Corlette class, are presented in *Table S2*. While right-sided and left-sided resections respectively were the norm for Bismuth–Corlette type IIIa and IIIb tumours at both centres, the extent of resection differed, with trisectionectomies being performed more frequently at Karolinska: *n* = 38, 80.9% *versus n* = 1, 2.5% at CIH for IIIa tumours (*P* < 0.001) and *n* = 6, 42.9% *versus n* = 3, 7.1% for IIIb tumours (*P* = 0.005).

For Bismuth–Corlette type IV tumours, the side of resection differed, with left hepatectomies more common at CIH and right hepatectomies more common at Karolinska (*P* = 0.002).

While the rate of a positive proximal or distal margin after resection of Bismuth–Corlette type I and II tumours was similar between centres, there was a marked difference in radial R1 (*n* = 3, 6.7% at CIH *versus n* = 5, 50.0% at Karolinska, *P* = 0.003, *Table S3*). In patients with Bismuth–Corlette type IIIa, IIIb and IV tumours, no significant differences in positive margin rates were observed between centres. When comparing R1 by T-status, similar rates were found at both centres in T3 and T4 tumours. In patients with T1 and T2 tumours, R1 at the radial margin was more frequent in the Karolinska cohort, while distal R1 was more frequent in the CIH cohort (*Table S3*).

### Short-term postoperative outcomes

The rate of postoperative complications Clavien–Dindo grade ≥ IIIa was 24.5% at CIH and 51.1% at Karolinska (*P* < 0.001). Rates of PHLF grades B–C and bile leak grades B–C were both significantly lower at CIH (PHLF 7.5% at CIH *versus* 25.6% at Karolinska, *P* < 0.001; bile leak 18.9% at CIH *versus* 31.1% at Karolinska, *P* < 0.001). The postoperative 90-day mortality rate was 2.5% at CIH *versus* 10.0% at Karolinska (*P* = 0.016). Causes of death are presented in *Table S4*. Grade C PHLF was the cause of death in *n* = 11, 84.6% of deaths (CIH *n* = 3, 75.0%, Karolinska *n* = 8, 88.9%). Among patients with postoperative death after PHLF grade C (*n* = 11), liver failure was frequently aggravated by postoperative bleeding (*n* = 5, 45.5%).

Preoperative ASA, FLR and extended resections were univariably associated with the mortality rate in the joint bi-institutional cohort (*Table S5*). Factors associated with the rate of complications Clavien-Dindo grade IIIa or above were: extended resection, BMI, preoperative percutaneous biliary drainage and preoperative FLR (*Table S5*). The rate of postoperative abscess was similar at both institutions (CIH *n* = 49, 30.8%, Karolinska *n* = 26, 29.5%, *P* = 0.835, missing data abscess *n* = 2), and no association was seen between type of biliary drainage and risk of abscess (percutaneous transhepatic biliary drainage *n* = 13, 30.2%, endoscopic biliary drainage *n* = 54, 35.3%, *P* = 0.536).

### Survival analysis

**Fig. 1:**
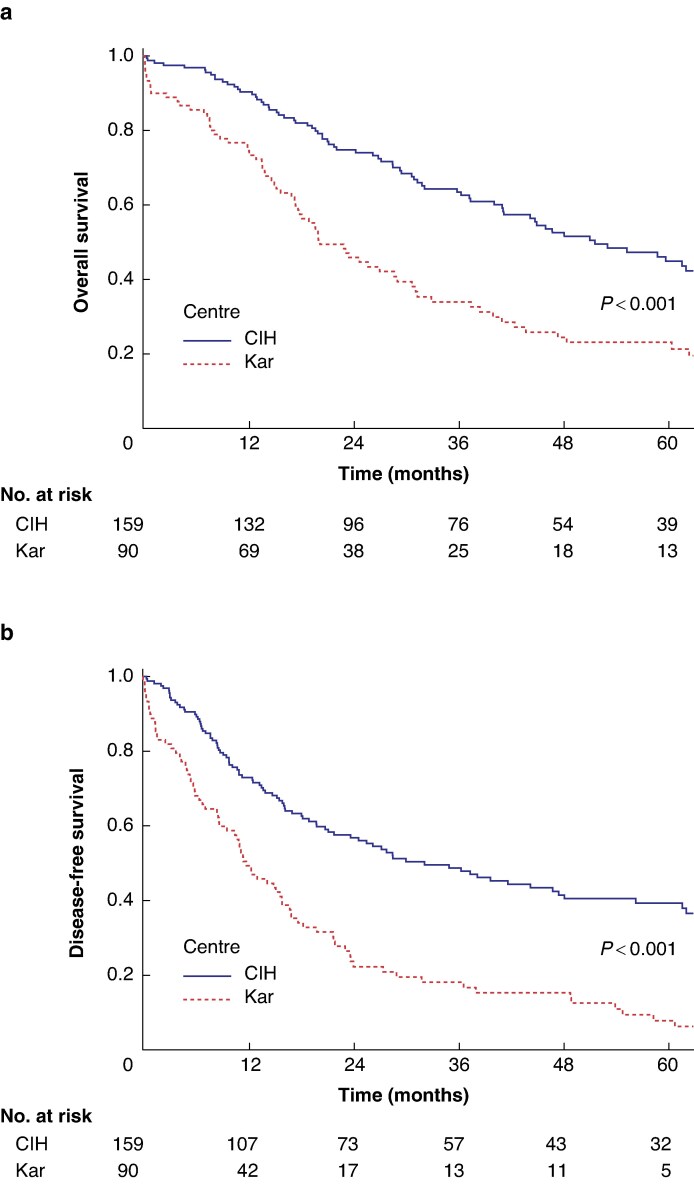
a Overall survival and b disease-free survival after resection for pCCA with stratification according to centre CIH, Cancer Institute Hospital; Kar, Karolinska University Hospital; pCCA, perihilar cholangiocarcinoma.

Overall survival is presented in *Fig. 1a* and DFS in *Fig. 1b*. Median OS was 20.4 months (95% c.i. 14.6 to 26.1) for patients at Karolinska and 52.0 months (95% c.i. 39.3 to 64.8) for patients at CIH (*P* < 0.001). Median DFS was 11.9 months (95% c.i. 8.6 to 15.2) at Karolinska and 32.4 months (95% c.i. 20.1 to 44.7) at CIH (*P* < 0.001). Differences in OS and DFS remained when excluding patients with postoperative fatality (median OS 24.9 months Karolinska *versus* 53.5 months CIH, *P* < 0.001; median DFS 14.3 months Karolinska *versus* 35.2 months CIH, *P* < 0.001, *Fig. S1a,b*).

**Fig. 2:**
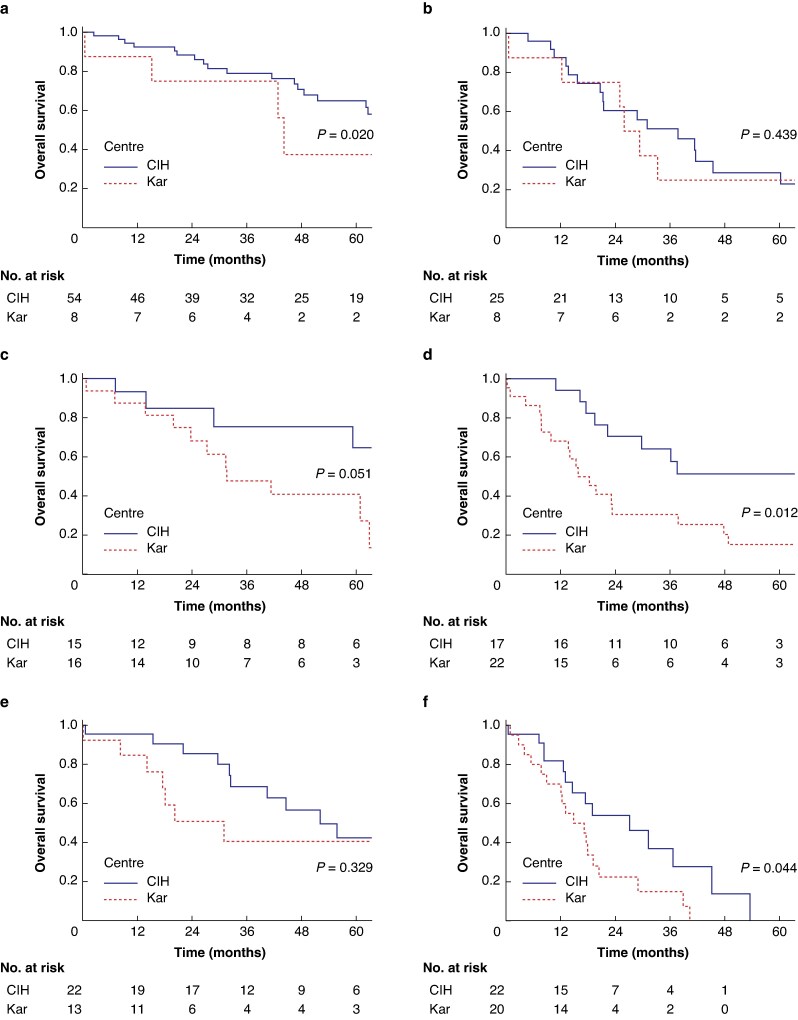
Overall survival after resection for pCCA according to tumour extension and lymph node status **a** T1–T2a N0, **b** T1–T2a N1, **c** T2b N0, **d** T2b N1, **e** T3–T4 N0, **f** T3–T4 N1. CIH, Cancer Institute Hospital; Kar, Karolinska University Hospital; N0/N1, absence/presence of lymph node metastasis; pCCA, perihilar cholangiocarcinoma; T, tumour extension status.

Overall survival differed between centres within each T-status subgroup (T2a: *P* = 0.013; T2b: *P* = 0.003; T3: *P* = 0.033; T4: *P* < 0.001). Survival curves separated by T-status and N-status are presented in *Fig. 2a–f*. The most pronounced differences in long-term survival were seen in earlier tumour stages (*Fig. 2a,d*) with the exception of the T1–T2a N1 subgroup (*Fig. 2b*), while for patients with T3 and T4 tumours long-term OS outcomes were poor at both centres (*Fig. 2e–f*), with no 5-year postoperative survival at either centre in the presence of lymph node metastasis (*Fig. 2f*). If excluding patients with postoperative fatality, the comparisons between centres for OS according to T- and N-status remained similar, while the difference for T1–T2a N0 did not reach statistical significance (*P* = 0.056) (*Fig. S2a–f*). As adjuvant therapy was introduced systematically only from 2019 at the Western centre, a subanalysis of OS and DFS was performed for the intervals 2010–2018 and 2019–2022 respectively. No significant differences were seen in OS (*P* = 0.682) or DFS (*P* = 0.201) (*Fig. S3a,b*).

### Uni- and multivariable Cox regression analysis

Factors associated with OS at each centre and in the overall bi-institutional cohort were analysed by Cox regression (*Table 2*). In multivariable analysis, both after variable selection and in a full model (*Table 2*; *Table S6*), treatment centre, age, T-status, N-status, CA19-9 and a positive resection margin were independently associated with survival. In the full model (*Table S6*), a negative association with survival was also observed for right-sided resection (HR 1.56, 95% c.i. 1.03 to 2.37, *P* = 0.036). Sensitivity analyses are presented in *Table S7* including complete case analysis and adjustment for PSC status, with similar results and the same factors identified as significant. PSC was not associated with survival on univariable (HR 1.61, 95% c.i. 0.87 to 2.99) or multivariable analysis (*Table S7*).

**Table 2:** Uni- and multivariable Cox regression analyses for overall survival

	CIH, univariable HR (95% c.i.)	*P*	Karolinska, univariable HR (95% c.i.)	*P*	All, multivariable HR (95% c.i.)	*P*
Centre (CIH)	–	–	–	–	**0.46** **(0.31,0.67)**	**<0.001**
Age (years)	**1.03** **(1.01,1.05)**	**0.016**	**1.01** **(1.00,1.03)**	**0.112**	**1.02** **(1.01,1.04)**	**0.005**
Sex (male)	1.32 (0.77,2.26)	0.309	1.29 (0.81,2.05)	0.287	–	–
BMI	0.98 (0.91,1.05)	0.509	1.01 (0.96,1.07)	0.656	–	–
ASA ≥ III	1.23 (0.68,2.24)	0.497	1.12 (0.68,1.85)	0.645	–	–
CA19-9 (U/ml)	**1.00** **(1.00,1.00)**	**<0.001**	**1.00** **(1.00,1.00)**	**0.044**	**1.00** **(1.00,1.00)**	**0.017**
PVE	1.18 (0.75,1.86)	0.471	1.36 (0.84,2.21)	0.345	–	–
Right-sided resection	1.31 (0.84,2.05)	0.231	1.20 (0.73,1.96)	0.480	–	–
Extended resection	1.06 (0.59,1.93)	0.837	1.37 (0.82,2.30)	0.227	–	–
T ≥ 3	**1.68** **(1.04,2.71)**	**0.033**	**1.45** **(0.90,2.33)**	**0.129**	**1.68** **(1.17,2.41)**	**0.005**
N1	**2.31** **(1.46,3.65)**	**<0.001**	**1.77** **(1.09,2.86)**	**0.021**	**1.98** **(1.39,2.81)**	**<0.001**
Grade (≥2)	**1.61** **(0.99,2.61)**	**0.056**	**3.90** **(0.55,27.68)**	**0.173**	1.55 (0.97,2.49)	0.067
R1	**2.57** **(1.63,4.07)**	**<0.001**	**2.14** **(1.34,3.41)**	**0.001**	**2.14** **(1.52,3.01)**	**<0.001**
Adjuvant therapy*	0.99 (0.97,1.01)	0.366	1.00 (0.98,1.01)	0.583	–	–

Factors with *P* < 0.20 at univariable analysis or *P* < 0.05 at multivariable analysis in bold type. ASA ≥ III, American Society of Anesthesiologists physical status class 3 or above; BMI, body mass index; CA19-9, carbohydrate antigen 19-9; CIH, Cancer Institute Hospital; Grade ≥ II, moderate or poor tumour differentiation; N1, lymph node metastasis; PVE, portal vein embolization; T ≥ 3, tumour extension status 3 or 4; R1, microscopically tumour positive margin. *As time-dependent variable.

## Discussion

In this cohort, important differences in tumour characteristics and surgical strategies for resectable pCCA were identified between a Western and an Eastern expert centre. Disparities in OS persisted across centres when analysed by tumour extension and lymph node status. In contrast to previous comparative studies^1,8^, important factors such as radial margin status and use of neoadjuvant/adjuvant therapy were evaluated. No differences were seen in the use of perioperative chemotherapy, whereas more advanced tumours and radial margin positivity were found to contribute to poor long-term survival at the Western centre. The burden of comorbidity, extent of resection and remnant liver volume were identified as risk factors for death.

Despite more advanced tumours and extended resections at the Western centre, PVE and left-sided resections were more frequent at the Eastern centre. While the 90-day mortality rate in the Western cohort was below a recently proposed international benchmark^1^, it was four-fold compared with the Eastern counterpart. Higher ASA class combined with smaller FLR impacted the Western fatality risk.

During the study period, the Eastern centre routinely performed volumetry, employing a target FLR of ≥40%, in contrast to the Western centre that employed a target ≥30% and predominantly performed volumetry and PVE before extended right-sided resections. In the Eastern cohort, a larger FLR was reached through more frequent PVE, more frequent use of left-sided resection and cautious use of trisectionectomy, which was mainly reserved for Bismuth–Corlette class IV tumours.

Radial margin positivity was more frequent in the Western cohort. The higher proportion of T2b tumours in the Western cohort indicates how radial R1 can be considered a surrogate for more advanced T-status. Distal margin positivity was more prevalent in the Eastern cohort, suggesting the possibility of more longitudinally extended tumours.

The differences in demography and tumour characteristics suggest that variations in tumour biology may contribute to disparities in long-term outcomes. Despite less extensive lymphadenectomy, the Western cohort showed a higher incidence of lymph node metastasis. While the extent of lymphadenectomy has been suggested to affect risk of recurrence^27^, its primary purpose is accurate staging^28,29^. The Western N0 group may include patients who would have been upstaged to N1 with more extensive lymph node sampling.

Multivariable analysis showed a negative association between right-sided resections and OS, consistent with recent reports indicating superior outcomes with left-sided resections^1,30,31^. The oncological radicality of left-sided resection has also been demonstrated in advanced pCCA^32–36^.

These results suggest that disparities in long-term outcomes result from both biological variation and differences in management. The degree to which differences in tumour characteristics represent patients diagnosed later with more advanced disease in the Western cohort, or underlying geographical differences in disease biology, remains to be determined. Established differences in CCA incidence and risk factors between Eastern and Western countries^37,38^ imply that clinical characteristics could be expected to diverge. In the previous comparison by Kimura *et al*., a more advanced stage and higher R1 rate was reported in the Western cohort, while moderately differentiated tumours were less frequent^9^. In the study from Olthof *et al*., patients in the Western series had lower rates of perineural and lymphovascular invasion, and R1 rates were similar^8^. To assess the role of patient selection and referral patterns, all-comer studies and population-based analyses are mandated.

The use of perioperative chemotherapy did not differ. Until recent phase III data^6,7^, only selected patients with high-risk characteristics received adjuvant therapy at both institutions. The role of neoadjuvant therapy in biliary tract cancer remains to be defined^39,40^. Given the poor long-term survival in T3–T4 pCCA with lymph node metastasis seen here, with no patient reaching 5-year survival, pCCA-specific neoadjuvant trials would seem motivated.

This study had several limitations. Due to its observational and retrospective nature, missing data and the risk of bias or residual confounding could not be avoided. The sample size restricted the number of covariates in multivariable analysis. The time from initial diagnosis to surgery, which could affect OS, was not available. While both institutions registered the same resection margins, no common re-assessment was attainable. The impact of preoperative ASA class on the mortality rate underscores the importance of further analysis of frailty in pCCA, using standardized assessments of physical function^41–43^. While providing granular data, this bi-institutional analysis did not describe wider regional trends or tendencies.

In conclusion, notable differences in short- and long-term outcomes were identified, with a benefit for patients in the Eastern cohort. More advanced tumours contributed to poor long-term survival in the Western cohort, together with more frequent radial margin positivity. A higher comorbidity burden and a higher rate of extended resections with smaller FLR influenced the Western postoperative mortality rate. Modifiable factors such as type of resection and preoperative interventions are crucial in minimizing the postoperative morbidity rate. Within the limits of tumour biology, an individualized surgical strategy and optimized patient selection can substantially impact long-term outcomes for patients with pCCA.

## Supplementary Material

zraf019_Supplementary_Data

## Data Availability

The data that support the findings of this study are available from the corresponding author upon reasonable request.
