# Supplementary material for: Western and Eastern experience in treating perihilar cholangiocarcinoma: retrospective bi-centre study
Source: BJS Open. 2025 Apr 9;9(2):zraf019. doi: 10.1093/bjsopen/zraf019 (PMC11979329; doi:10.1093/bjsopen/zraf019)
Supplement: zraf019_Supplementary_Data [file zraf019_supplementary_data.docx]

**At the Crossroads: Perihilar Cholangiocarcinoma in the Current Western and East-ern Experience – Different Strategies for Similar Tumours?**

Authors: (* Shared first authorship; # Shared supervisors)

Hannes Jansson, MD, PhD ^a,b^*, Atsushi Oba, MD, PhD ^b^*, Aya Maekawa, MD, PhD ^b^, Christina Villard, MD, PhD ^c^, Kosuke Kobayashi, MD, PhD ^b^, Yoshihiro Ono, MD, PhD ^b^, Jennie Engstrand, MD, PhD ^a^, Fumihiro Kawano, MD, PhD ^b^, Hiromichi Ito, MD ^b^, Stefan Gilg, MD, PhD ^a^, Yosuke Inoue, MD, PhD ^b^, Melroy A D’Souza, MD, PhD ^a#^, Yu Takahashi, MD, PhD ^b#^

Affiliations:

^a^ Division of Surgery and Oncology, Department of Clinical Science, Innovation and Technology, Karolinska Institutet, Stockholm, Sweden

^b^ Division of Hepatobiliary and Pancreatic Surgery, Cancer Institute Hospital, Japanese Foundation for Cancer Research, Tokyo, Japan

^c^ Division of Transplantation Surgery, Department of Clinical Science, Innovation and Technology, Karolinska Institutet, Stockholm, Sweden

**Corresponding author:**

Yu Takahashi, MD PhD

Division of Hepatobiliary and Pancreatic Surgery, Cancer Institute Hospital

Japanese Foundation for Cancer Research

3-8-31 Ariake, Koto-ku, Tokyo 135-8550, Japan

E-mail: yu.takahashi@jfcr.or.jp **ORCID ID:** 0000-0003-3066-023X

**Supplementary Materials - Index**

| **Supplementary Figures and Tables** |  |
| --- | --- |
| Supplementary Figure 1A-B | *page 2* |
| Supplementary Figure 2A-F | *page 3* |
| Supplementary Figure 3A-B | *Page 4* |
| Supplementary Table 1 | *page 5* |
| Supplementary Table 2 | *page 6* |
| Supplementary Table 3 | *page 7* |
| Supplementary Table 4 | *page 8* |
| Supplementary Table 5 | *page 9* |
| Supplementary Table 6 | *page 10* |
| Supplementary Table 7 | *page 11* |

**Supplementary Figure 1A-B.** Overall survival (A) and disease-free survival (B), excluding postoperative 90-day mortality, after resection for pCCA with stratification according to centre (blue: CIH; green: Karolinska)

CIH: Cancer Institute Hospital; Kar: Karolinska University Hospital; pCCA: perihilar cholangiocarcinoma.

**Supplementary Figure 2A-F.** Overall survival after resection for pCCA, excluding postoperative 90-day mortality, according to tumour extension and lymph node status, A: T1-T2a N0; B: T1-T2a N1; C: T2b N0; D: T2b N1; E: T3-T4 N0; F: T3-T4 N1

CIH: Cancer Institute Hospital; Kar: Karolinska University Hospital; N0/N1: absence/presence of lymph node metastasis; pCCA: perihilar cholangiocarcinoma; T: tumour extension status.

**Supplementary Figure 3A-B.** Overall survival (A) and disease-free survival (B) after resection for pCCA in the Western cohort, with stratification according to time period (2010-2018 blue line, 2019-2022 green line)

pCCA: perihilar cholangiocarcinoma.

**Supplementary Table 1.** Lymph node metastasis and tumour differentiation according to tumour extension

|  | **CIH, n (%)** | **Karolinska, n (%)** | **P-value** |
| --- | --- | --- | --- |
| **Lymph node metastasis** |  |  |  |
| **T1-T2a N1** | 25 (31.6) | 8 (50.0) | 0.160 |
| **T2b N1** | 17 (53.1) | 22 (57.9) | 0.689 |
| **T3-T4 N1** | 22 (50.0) | 20 (60.6) | 0.355 |
| **Differentiation** |  |  |  |
| **T1-T2a Grade ≥2** | 48 (63.2) | 16 (100) | **0.002^#^** |
| **T2b Grade ≥2** | 22 (68.8) | 37 (94.9) | **0.003** |
| **T3-T4 Grade≥2** | 29 (67.4) | 31 (96.9) | **0.002** |

Grade≥2: moderate or poor tumour differentiation; N1: lymph node metastasis; T: tumour extension status.

# Fisher’s Exact test

**Supplementary Table 2.** Resection types according to Bismuth-Corlette class

| **Group, n per centre** | **LH, n (%)** | **LT, n (%)** | **RH, n (%)** | **RT, n (%)** | **CB/other, n (%)** | **P-value^#^** |
| --- | --- | --- | --- | --- | --- | --- |
| **BC I** |  |  |  |  |  |  |
| **CIH n=20** | 6 (30.0) | 0 | 14 (70.0) | 0 | 0 | **0.004** |
| **Kar n=3** | 1 (33.3) | 0 | 0 | 1 (33.3) | 1 (33.3) |  |
| **BC II** |  |  |  |  |  |  |
| **CIH n=25** | 10 (40.0) | 0 | 13 (52.0) | 0 | 2 (8.0) | **0.003** |
| **Kar n=7** | 2 (28.6) | 1 (14.3) | 1 (14.3) | 3 (42.9) | 0 |  |
| **BC IIIa** |  |  |  |  |  |  |
| **CIH n=40** | 0 | 1 (2.5) | 38 (95.0) | 0 | 1 (2.5) | **<0.001** |
| **Kar n=47** | 1 (2.1) | 2 (4.3) | 8 (17.0) | 36 (76.6) | 0 |  |
| **BC IIIb** |  |  |  |  |  |  |
| **CIH n=42** | 38 (90.5) | 3 (7.1) | 0 | 0 | 1 (2.4) | **0.005** |
| **Kar n=14** | 8 (57.1) | 6 (42.9) | 0 | 0 | 0 |  |
| **BC IV** |  |  |  |  |  |  |
| **CIH n=32** | 7 (21.9) | 17 (53.1) | 4 (12.5) | 3 (9.4) | 1 (3.1) | **0.002** |
| **Kar n=12** | 2 (16.7) | 2 (16.7) | 0 | 8 (66.7) | 0 |  |

BC: Bismuth-Corlette class; CIH: Cancer Institute Hospital; Kar: Karolinska University Hospital; CB: central bisectionectomy (H148-B/H48-B); LH: left hemihepatectomy (H1234-B/H234-B); LT: left trisectionectomy (H123458-B/H23458-B); RH: right hemihepatectomy (H15678-B/H5678-B); RT: right trisectionectomy (H145678-B/H45678-B).

# Fisher’s Exact test

**Supplementary Table 3.** Radicality of resections according to Bismuth-Corlette class and tumour extension

| **Group, n per centre** | **R1 proximal**  **n (%)** | **P-value** | **R1 distal**  **n (%)** | **P-value** | **R1 radial**  **n (%)** | **P-value** |
| --- | --- | --- | --- | --- | --- | --- |
| **BC I-II** |  |  |  |  |  |  |
| **CIH n=45** | 13 (28.9) | 0.423^#^ | 10 (22.2) | 0.667^#^ | **3 (6.7)** | **0.003^#^** |
| **Kar n=10** | 1 (10.0) |  | 1 (10.0) |  | **5 (50.0)** |  |
| **BC IIIa** |  |  |  |  |  |  |
| **CIH n=39** | 7 (17.9) | 0.946 | 7 (17.9) | 0.073^#^ | 4 (10.5) | 0.347 |
| **Kar n=46** | 8 (17.4) |  | 2 (4.3) |  | 8 (17.4) |  |
| **BC IIIb** |  |  |  |  |  |  |
| **CIH n=41** | 6 (14.6) | 1.000^#^ | 4 (9.8) | 0.562^#^ | 2 (4.9) | 0.265^#^ |
| **Kar n=14** | 2 (14.3) |  | 0 |  | 2 (14.3) |  |
| **BC IV** |  |  |  |  |  |  |
| **CIH n=32** | 5 (15.6) | 0.227^#^ | 7 (21.9) | 0.163^#^ | 8 (25.0) | 0.707^#^ |
| **Kar n=12** | 4 (33.3) |  | 0 |  | 4 (33.3) |  |
| **T1-T2** |  |  |  |  |  |  |
| **CIH n=110** | 22 (20.0) | 0.741 | **20 (18.2)** | **<0.001** | **5 (4.5)** | **0.007^#^** |
| **Kar n=54** | 12 (22.2) |  | **0** |  | **10 (18.5)** |  |
| **T3-T4** |  |  |  |  |  |  |
| **CIH n=44** | 7 (15.9) | 0.746^#^ | 7 (15.9) | 0.746^#^ | 12 (27.3) | 0.898 |
| **Kar n=35** | 4 (11.4) |  | 4 (11.4) |  | 10 (28.6) |  |

CIH: Cancer Institute Hospital; Kar: Karolinska University Hospital; R1: microscopically tumour positive margin; T1-T2: tumour extension status 1 or 2; T3-T4: tumour extension status 3 or 4.

# Fisher’s Exact test

**Supplementary Table 4.** Postoperative 90-day mortality

|  | **Complications, descriptive** | **Resection type** |
| --- | --- | --- |
| **CIH (n=4)** | Paralytic ileus, liver failure, renal failure | RH |
|  | Postoperative bleeding, liver failure | RH |
|  | Postoperative bleeding, liver failure | RH |
|  | Pneumonia, respiratory failure | LH |
| **Karolinska (n=9)** | Postoperative bleeding, liver failure | RT |
|  | Liver failure | RT |
|  | Liver failure, renal failure | RH |
|  | Postoperative bleeding, liver failure | LH |
|  | Liver failure, renal failure | LH |
|  | Sepsis, cardiac arrest | RT |
|  | Liver failure, renal failure | RT |
|  | Postoperative bleeding, liver failure, heart failure | LT |
|  | Liver failure, bleeding complications | RT |

CIH: Cancer Institute Hospital; Karolinska: Karolinska University Hospital; LH: left hemihepatectomy (H1234-B/H234-B); LT: left trisectionectomy (H123458-B/H23458-B); RH: right hemihepatectomy (H15678-B/H5678-B); RT: right trisectionectomy (H145678-B/H45678-B).

**Supplementary Table 5.** Univariable associations with postoperative morbidity and mortality within the joint bi-institutional cohort

|  | **Clavien-Dindo grade ≥IIIa** | | **P-value** | **90-day mortality** | | **P-value** |
| --- | --- | --- | --- | --- | --- | --- |
|  | **No n=164** | **Yes n=85** |  | **No n=236** | **Yes n=13** |  |
| **Age, years, md (IQR)** | 68 (60-74) | 68 (58-73) | 0.382 | 68 (58-74) | 69 (60.5-73) | 0.876 |
| **Sex (male), n (%)** | 106 (64.6) | 61 (71.8) | 0.256 | 157 (66.5) | 10 (76.9) | 0.554^#^ |
| **BMI, md (IQR)** | 21.9 (20.2-24.4) | 23.5 (20.9-27.2) | **0.012** | 22.3 (20.4-25.1) | 24.1 (22.3-28.9) | 0.088 |
| **ASA≥3, n (%)** | 33 (20.2) | 22 (25.9) | 0.310 | 49 (20.9) | 6 (46.2) | **0.043^#^** |
| **PTBD, n (%)** | 23 (14.0) | 21 (24.7) | **0.036** | 40 (16.9) | 4 (30.8) | 0.254^#^ |
| **PVE, n (%)** | 77 (47.0) | 40 (47.1) | 0.987 | 109 (46.2) | 8 (61.5) | 0.280 |
| **FLR, percent, md (IQR)** | 46.4 (39.5-62.8) | 39.2 (28.2-53.4) | **<0.001** | 45.2 (36.5-59.1) | 35.0 (21.6-45.2) | **0.029** |
| **Right-sided resection, n (%)** | 79 (48.2) | 51 (60.0) | 0.076 | 122 (51.7) | 8 (61.5) | 0.489 |
| **Extended resection, n (%)** | 44 (26.8) | 40 (47.1) | **0.001** | 76 (32.2) | 8 (61.5) | **0.037^#^** |

ASA≥3: American Society of Anesthesiologists physical status class 3 or above; BMI: body mass index; FLR: future liver remnant; PTBD: percutaneous biliary drainage; PVE: portal vein embolization.

# Fisher’s Exact test

**Supplementary Table 6.** Multivariable Cox regression analysis of overall survival, full model including all variables from univariable analysis (bold type: P<0.05)

|  | **CIH,**  **univariable**  **HR (95% CI)** | **P-value** | **Karolinska,**  **univariable**  **HR (95% CI)** | **P-value** | **All, multivariable**  **HR (95% CI)** | **P-value** |
| --- | --- | --- | --- | --- | --- | --- |
| **Center (CIH)** |  |  |  |  | **0.51**  **(0.29-0.89)** | **0.018** |
| **Age (years)** | **1.03**  **(1.01-1.05)** | **0.016** | 1.01  (1.00-1.03) | 0.112 | **1.02**  **(1.00-1.04)** | **0.012** |
| **Sex (male)** | 1.32  (0.77-2.26) | 0.309 | 1.29  (0.81-2.05) | 0.287 | 1.35  (0.93-1.95) | 0.110 |
| **BMI** | 0.98  (0.91-1.05) | 0.509 | 1.01  (0.96-1.07) | 0.656 | 1.00  (0.95-1.04) | 0.839 |
| **ASA≥3** | 1.23  (0.68-2.24) | 0.497 | 1.12  (0.68-1.85) | 0.645 | 1.05  (0.67-1.63) | 0.845 |
| **CA19-9 (U/mL)** | **1.00**  **(1.00-1.00)** | **<0.001** | **1.00**  **(1.00-1.00)** | **0.044** | **1.00**  **(1.00-1.00)** | **0.004** |
| **PVE** | 1.18  (0.75-1.86) | 0.471 | 1.36  (0.84-2.21) | 0.345 | 0.90  (0.57-1.41) | 0.641 |
| **Right-sided resection** | 1.31  (0.84-2.05) | 0.231 | 1.20  (0.73-1.96) | 0.480 | **1.56**  **(1.03-2.37)** | **0.036** |
| **Extended resection** | 1.06  (0.59-1.93) | 0.837 | 1.37  (0.82-2.30) | 0.227 | 1.16  (0.76-1.78) | 0.482 |
| **T≥3** | **1.68**  **(1.04-2.71)** | **0.033** | **1.45**  **(0.90-2.33)** | 0.129 | **1.93**  **(1.30-2.87)** | **0.001** |
| **N1** | **2.31**  **(1.46-3.65)** | **<0.001** | **1.77**  **(1.09-2.86)** | **0.021** | **2.09**  **(1.46-2.99)** | **<0.001** |
| **Grade (≥2)** | 1.61  (0.99-2.61) | 0.056 | 3.90  (0.55-27.68) | 0.173 | 1.62  (1.00-2.62) | 0.052 |
| **R1** | **2.57**  **(1.63-4.07)** | **<0.001** | **2.14**  **(1.34-3.41)** | **0.001** | **2.15**  **(1.52-3.05)** | **<0.001** |
| **Adjuvant therapy ^§^** | 0.99  (0.97-1.01) | 0.366 | 1.00  (0.98-1.01) | 0.583 | 0.99  (0.97-1.00) | 0.055 |

ASA≥3: American Society of Anesthesiologists physical status class 3 or above; BMI: body mass index; CA19-9: carbohydrate antigen 19-9; CIH: Cancer Institute Hospital; Grade≥2: moderate or poor tumour differentiation; N1: lymph node metastasis; PVE: portal vein embolization; T≥3: tumour extension status 3 or 4; R1: microscopically tumour positive margin. §: As time-dependent variable.

**Supplementary Table 7.** Multivariable Cox regression of overall survival. Sensitivity analyses with complete case analysis; complete case analysis excluding CA19-9; adjustment for PSC status (bold type: P<0.05)

|  | **n=199, multivariable**  **HR**  **(95% CI)** | **P-value** | **n=232,**  **multivariable**  **HR**  **(95% CI)** | **P-value** | **n=199, multivariable**  **HR**  **(95% CI)** | **P-value** |
| --- | --- | --- | --- | --- | --- | --- |
| **Center (CIH)** | **0.48**  **(0.31-0.76)** | **0.001** | **0.43**  **(0.29-0.63)** | **<0.001** | **0.51**  **(0.32-0.81)** | **0.005** |
| **Age (years)** | 1.02  (1.00-1.03) | 0.072 | **1.02**  **(1.01-1.04)** | **0.006** | **1.02**  **(1.00-1.04)** | **0.033** |
| **CA19-9 (U/mL)** | **1.00**  **(1.00-1.00)** | **0.024** |  |  | **1.00**  **(1.00-1.00)** | **0.023** |
| **T≥3** | **1.65**  **(1.08-2.50)** | **0.019** | **1.77**  **(1.23-2.56)** | **0.002** | **1.67**  **(1.10-2.54)** | **0.017** |
| **N1** | **2.14**  **(1.40-3.28)** | **<0.001** | **2.35**  **(1.62-3.41)** | **<0.001** | **2.12**  **(1.37-3.26)** | **<0.001** |
| **Grade (≥2)** | 1.57  (0.98-2.52) | 0.063 | **1.60**  **(1.00-2.57)** | **0.049** | 1.58  (0.98-2.54) | 0.059 |
| **R1** | **1.84**  **(1.24-2.71)** | **0.002** | **1.99**  **(1.40-2.83)** | **<0.001** | **1.83**  **(1.24-2.70)** | **0.003** |
| **Adjuvant therapy ^§^** | 0.99  (0.97-1.00) | 0.101 | 0.99  (0.98-1.00) | 0.083 | 0.99  (0.97-1.00) | 0.071 |
| **PSC** |  |  |  |  | 1.75  (0.71-4.34) | 0.225 |

CA19-9: carbohydrate antigen 19-9; CIH: Cancer Institute Hospital; Grade≥2: moderate or poor tumour differentiation; N1: lymph node metastasis; PSC: primary sclerosing cholangitis; PVE: portal vein embolization; T≥3: tumour extension status 3 or 4; R1: microscopically tumour positive margin. §: As time-dependent variable.
